# Enabling Access to Pharmacy Law Teaching during COVID-19: Student Perceptions of MyDispense and Assessment Outcomes

**DOI:** 10.3390/pharmacy11020044

**Published:** 2023-02-24

**Authors:** Natasha Slater, Thuy Mason, Ricarda Micallef, Madhvee Ramkhelawon, Leanne May

**Affiliations:** Department of Pharmacy, Kingston University London, Kingston upon Thames, London KT1 2EE, UK

**Keywords:** MyDispense, pharmacy, pharmacy students, simulation, COVID-19

## Abstract

During the COVID-19 pandemic, it was not always possible to teach pharmacy practice and practical dispensing skills in person. Second-year pharmacy students (*n* = 147) were given access to a virtual simulation tool, MyDispense, to supplement their learning. This software enabled students to work remotely and access exercises in a ‘safe’ community pharmacy setting. This study evaluated second-year pharmacy students’ perceptions of MyDispense, and the impact on assessment performance in the pharmacy law and ethics module. Students were able to access 22 MyDispense activities throughout the academic year (2020/2021). Exercise completion rates and assessment marks were analysed, along with findings from a cross-sectional survey about user experience. MyDispense data were available for all students (*n* = 147), and 76.1% (*n* = 115) completed the survey. The mean number of MyDispense exercises completed was nine. Higher levels of MyDispense exercise completion were associated with higher pass rates and mean scores (no exercises completed versus 1–10 activities completed: mean score 77.1 versus 83.1, respectively) and a statistically significant association between exercise usage and in-class assessed prescription scores. Overall, 46.1% (*n* = 53/115), 33.0% (*n* = 38/115) and 33.9% (*n* = 39/115) of students felt that MyDispense had helped them to prepare for their assessed prescriptions, mid-module test, and final exam, respectively. MyDispense has provided an accessible alternative to in-person teaching for students during the COVID-19 pandemic, and results showed a positive association with assessment performance in pharmacy law and ethics.

## 1. Introduction

Prior to the coronavirus disease of 2019 (COVID-19) pandemic, online learning and virtual simulations were evolving rapidly and have now become fundamental to modern healthcare education [1,2,3]. During the COVID-19 pandemic educational institutions, including universities, had to quickly adapt and change their teaching methods in a limited amount of time [4]. Online learning and virtual simulations allow an adaptable approach to teaching, using a variety of resources [5,6]. Virtual simulations have been described as a space where students can practice their clinical skills during their training, without the risk of making mistakes that would negatively impact patient safety or result in guilt for the student [7,8]. It also allows students to be engaged in their learning by applying their pharmacy knowledge to real life scenarios [9].

As the role of the pharmacist continues to evolve, to become more clinically focused and service related, it is crucial to enhance student knowledge, particularly through simulation-based learning (SBL) [10]. Long-term knowledge retention has been shown to increase through SBL when compared to traditional learning methods [11].

When implemented into teaching previously, SBL has proven to be an effective method of learning, especially when integrated within the curriculum as it allows the students to repeatedly practice their skills in a controlled environment [12].

Coyne et al. [13] conducted a review that involved 23 studies that look into the use of virtual simulation to assess the clinical competency of healthcare students. Overall, this review showed that with virtual simulation, students’ confidence in their clinical skills was improved, especially because of the opportunity to practice their skills repeatedly before going into real situations. Another study has shown that students who used virtual simulation, strongly agreed or agreed (84%, *n* = 202) that virtual simulation allowed the students to safely make errors without causing patient harm [14]. Furthermore, SBL can be used to improve communication skills, build confidence, and reduce the number of errors leading to patient safety [10]. In addition, SBL offers learners a chance to apply their knowledge to simulated scenarios and practice the skills they had previously acquired in a didactic manner [15]. This allows the learners to put their knowledge into practice and obtain feedback to support their learning.

As part of the four-year Master of Pharmacy (MPharm) degree, second-year pharmacy students at Kingston University are enrolled in the module “Pharmacy Law, Ethics and Practice”, PY5020. This module is dedicated to teaching the legal aspects of dispensing, while also promoting safe dispensing practices. Within the module, practicals and workshops are timetabled to consolidate and build on the various concepts taught in lectures.

The delivery of teaching during the academic year 2020–2021 was impacted by the COVID-19 pandemic. In the “Pharmacy Law, Ethics and Practice” module, students received practical and workshop sessions on campus, with recorded asynchronous lectures and online feedback sessions between September and December 2020. However, due to another lockdown from January to April 2021, all sessions were taught online. To supplement our online teaching of law and ethics, the second-year pharmacy students were given access to an online pharmacy simulation tool, MyDispense (Monash University, Parkville, Victoria, Australia).

MyDispense was created by the Faculty of Pharmacy and Pharmaceutical Sciences at Monash University, Australia. At present, there are 186 pharmacy schools worldwide in partnership with MyDispense [16,17]; this includes 16 universities in the United Kingdom, of which Kingston University is one. Each institution has access to a wide variety of patient datasets and medications, thus enabling them to tailor the exercises for their students [18]. The popularity of MyDispense continues to rise, with over 25,000 students completing over 963,000 exercises in 2021 [19].

At Kingston University, the second-year pharmacy students were given access to MyDispense at the start of the academic year and were required to familiarise themselves with the layout of the virtual pharmacy community. Following this, the students were given prescription screening and dispensing exercises to complete. The students were required to receive and screen a prescription from their patient, choose the correct medication, label the medication appropriately, and supply the medication to their patient with counseling points.

MyDispense enabled the students to work remotely and virtually apply the principles of pharmacy law in a ‘safe’ pharmacy community setting, without compromising patient safety [20]. The virtual simulation tool also provided an opportunity for the students to learn from their mistakes by providing immediate and detailed feedback [21]. Previous research has shown that students felt that their dispensing skills had improved, in general, after using MyDispense, and they also felt more confident about their skills as they could practice dispensing in a safe environment [20].

To our knowledge, there has only been one study that has examined the impact of MyDispense usage on exam performance prior to this one [18]. Most studies have explored student perceptions of MyDispense [18,20,22,23]. Therefore, an inductive study was proposed seeking to add further data on the impact of using MyDispense to complement teaching.

The aim of this study was to evaluate second-year pharmacy students’ perceptions of MyDispense; in addition to examining the impact of MyDispense usage on assessment performance in the “Pharmacy Law, Ethics and Practice” module.

## 2. Materials and Methods

### 2.1. Study Design

A cross-sectional study was conducted. Student participation in MyDispense (Version 6.1.19 UK) activities’ data were analysed, for the academic year, and compared to module assessment performance data. In addition, students were asked to complete a cross-sectional survey to explore their perceptions and experiences of MyDispense.

To support students during the academic year 2020–2021, when the COVID-19 pandemic reduced the opportunity for face-to-face learning, 22 MyDispense exercises were created. There were 12 dispensing and 10 prescription validation activities. These activities mimicked the prescriptions that the students would have screened and dispensed if they had been receiving face-to-face teaching in the dispensing practical laboratory. The student completion data for the 22 activities were available on an “activity completion” report from MyDispense administration tools.

The 24-question evaluation survey was designed using a combination of sixteen closed and four Likert scale questions and four open-ended questions. The evaluation survey was broken into four sections covering student experience with MyDispense during the academic year, perceptions of the training provided through the use of MyDispense within the “Pharmacy Law, Ethics and Practice” module, and the impact on assessment preparation, and respondent demographics.

The exam and coursework (mid-module test and in-class assessed prescriptions) results for the “Pharmacy Law, Ethics and Practice”, PY5020, module were made available by the module leader at the end of the academic year.

### 2.2. Participants: Sampling and Recruitment

Human participants were used in this study. All second-year pharmacy students (*n* = 147) in the academic year 2020–2021 were encouraged to complete the MyDispense activities after each practical class. Activities were introduced during online feedback sessions, and links were provided to the students to allow them to subsequently complete the activities.

Respondents were asked to complete a cross-sectional survey to understand their experiences of using MyDispense. The respondents were emailed a survey link for completion of the survey on Microsoft Forms. A reminder email was sent after two weeks.

### 2.3. Data Collection

The completion data for the 22 MyDispense activities were downloaded as an activity completion report into Microsoft Excel. This report detailed each student’s attempt per activity. These data were then matched to the exam and coursework results, which were made available as a Microsoft Excel spreadsheet at the end of the academic year.

The second-year pharmacy student cohort were emailed a Microsoft Forms link inviting them to complete the survey. This email included a participant information sheet informing the student about the aim of the project and how their responses would be used. They were emailed in March 2021 after the module was finished to allow them time to reflect on their learning experience with MyDispense. An opportunity was given for questions to be raised if they wanted more information prior to completion of the survey. Implied consent of the participants was given on their completion of the survey and students were advised that they were under no obligation to participate and could withdraw up until the point of final submission.

### 2.4. Data Analysis

All survey responses were downloaded from Microsoft Forms to Microsoft Excel. All data were then transferred to IBM SPSS Statistics for Windows, Version 28.0 (2021), and coded for analysis. Open-ended questions were categorised in Microsoft Excel prior to coding in SPSS. Descriptive statistics, including percentages, frequencies, means, and modes were used, and Pearson correlation coefficients (r) were calculated. Correlations were described as weak (0–0.30), moderate (0.31–0.49), or strong (0.5–1) according to the r value [24]. Correlations were statistically significant at the 0.01 level (alpha).

The MyDispense exercise completion data were initially analysed in Microsoft Excel, and then matched to the “Pharmacy Law, Ethics and Practice” exam and coursework results (2021). According to the number of MyDispense activities they had completed during the academic year, students were allocated to one of the following four categories: none; 1–10; 11–22; and 23 or more. Assessment pass rates were also categorical variables. The association between MyDispense exercise completion rates and assessment pass rates was examined using a Chi-squared test. A *p*-value ≤ 0.05 was considered to be statistically significant. A one-way ANOVA was also performed to compare the effect of MyDispense activity usage on assessment marks.

### 2.5. Ethics

This study received ethical approval (1213/045) from the delegated ethical approval team operating under the ethics committee of the Kingston University Science, Engineering and Computing Faculty.

## 3. Results

MyDispense data were available for all second-year MPharm students (*n* = 147), and 78.2% (*n* = 115) completed a cross-sectional survey at the end of the academic year. The modal age range of students was 18–25 years.

### 3.1. MyDispense Usage and User Experience

There were 22 MyDispense exercises available throughout the academic year. The mean number of MyDispense exercises completed was nine. Over half of the surveyed students (59.1%, *n* = 68/115) reported that they had completed all the activities during the academic year.

The students were asked, “what have you enjoyed the most about using MyDispense during this academic year?” The response rate for this question was 71.3% (*n* = 82); their answers are presented in Table 1.

The students were also asked, “how could MyDispense be improved for future second-year MPharm students?” The response rate for this question was slightly lower than the previous question (61.7%, *n* = 71); their answers are presented in Table 2.

The MyDispense exercises included in this study enabled the students to develop their prescription screening, labeling, and product selection skills. However, 80.0% (*n* = 92/115) of the students surveyed were not aware that responding-to-symptoms (RTS) activities could also be created using MyDispense. Most students (82.6%, *n* = 95/115) reported that the RTS activities would be beneficial when preparing for their Objective Structured Clinical Examinations (OSCE) in subsequent years. Childhood conditions, headaches, respiratory infections, and ear conditions were identified as the preferred topics for future RTS activities on MyDispense. This study focused on the implementation of MyDispense among second-year students; however, 40.0% of the students surveyed (*n* = 46/115) would like to have MyDispense activities integrated into their third year of the MPharm course too.

Finally, the students were asked to suggest potential changes that could be made to MyDispense to reflect changes in pharmacy practice. The response rate for this question was 38.7% (*n* = 44); their answers are presented in Table 3.

### 3.2. Assessment Preparation

The students were asked if the MyDispense exercises had been helpful for their assessment preparation, in the “Pharmacy Law, Ethics and Practice” module. Overall, 46.1% (*n* = 53) of the students felt that MyDispense helped them to prepare for their assessed prescriptions, while 33.0% (*n* = 38) and 33.9% (*n* = 39) of the students felt that MyDispense had helped them to prepare for their mid-module test and final exam, respectively (Table 4).

Students were allocated into four groups according to the number of MyDispense exercises they had completed within the academic year. Higher levels of MyDispense exercise completion were associated with higher pass rates; however, findings were not statistically significant (Table 5).

A one-way ANOVA was also performed to compare the effect of MyDispense usage on assessment pass marks. Students who completed more MyDispense activities during the academic year achieved higher mean scores in the assessed prescription (*p* = 0.05). However, no statistically significant association was found between exam scores, or mid-module test scores, and MyDispense usage (Table 6).

Finally, a bivariate model was used to calculate Pearson’s correlation coefficients (*r*). A weak positive correlation (*r* = 0.24, *p* = 0.03) was detected between MyDispense usage and assessed prescription scores. However, no correlation was found between MyDispense usage and final exam scores (*r* = 0.06, *p* = 0.48) or MyDispense usage and mid-module test scores (*r =* −0.02, *p* = 0.82).

## 4. Discussion

Our study identified one statistically significant result which was an association between in-class assessed prescription mean score performance and MyDispense exercises completed during the academic year. Furthermore, a weak positive correlation was detected between MyDispense usage and assessed prescription scores. During the in-class assessed prescriptions, the students are required to legally and clinically screen a single-item prescription and dispense this, with correct labeling and product selection, in a physically simulated dispensary. Therefore, it is possible that this study found significance because the activities previously completed through MyDispense mimic the types of activities required during their assessment.

Higher levels of MyDispense exercise completion were also associated with higher pass rates and mean scores in exam and mid-module tests, although these findings were not statistically significant. Findings for these elements are supported by Shin et al. [18]. The latter study showed that MyDispense positively impacted exam scores, although their study also found that the differences were not statistically significant. A further study, looking at an increased use of blended learning in pharmacy law and ethics teaching, additionally failed to see a statistically significant improvement in student assessment performance [25]. Another study also showed that the higher use of online learning was associated with higher marks in module exams in a range of subjects; however, again no statistically significant difference was found [26].

### 4.1. Positives of MyDispense

MyDispense was highly used by the second-year pharmacy students during the academic year (2020–2021), and many benefits of the software were identified. Our students appreciated the accessibility of the exercises, the opportunity to repeat exercises, and the immediate provision of feedback; they also liked the realistic and interactive nature of the software. Ambroziak et al. [22] reported similar findings when they introduced the MyDispense software to first-year pharmacy students. Their students felt that MyDispense was an effective tool to develop dispensing skills in a classroom environment, and most of the students perceived MyDispense to be beneficial for their learning, particularly in relation to identifying and preventing medication errors [22]. This is consistent with another study which found 78% of the pharmacy students surveyed liked that they were able to practice dispensing in an environment where errors could be made without consequence [27]. Deneff et al. [23] also incorporated MyDispense into their teaching. Over a two-year period, fifty students were surveyed to explore their perceptions of MyDispense, which they had been used predominantly to screen prescriptions. At the end of this study, they concluded that the students generally found MyDispense to be a useful component in their pharmacy law and ethics learning; thus, their findings support the findings from the current study. Finally, our study has shown that students perceived MyDispense as helpful for assessment preparation, echoing previous studies using online learning environments [28,29]. As MyDispense predominantly supports the legal and clinical evaluation of prescriptions and their dispensing, it is not a surprise that the students perceived MyDispense as most helpful in preparing for their assessed prescription assignments. This is supported by our statistically significant finding in relation to assessed prescription performance improving with MyDispense usage. However, the concepts were also deemed to be helpful in formal test and exam situations, where law and ethical decision-making would be tested. Several students acknowledged the benefit of MyDispense to allow simulation, which was essential during the COVID-19 pandemic when access to experiential learning was more limited. Although face-to-face teaching occurred, students were unable to attend community pharmacy placements due to the COVID-19 restrictions imposed in healthcare settings in England.

### 4.2. Improvements Suggested

Despite identifying many benefits of MyDispense, our students felt that there were several opportunities for improvement. One common suggestion was that more instructions could be provided prior to use, while another suggestion was that the layout of the virtual simulation tool could be improved. Other improvements requested were a wider variety of exercises incorporating drug interactions and more patient interactions and different prescription types. Shawaqfeh et al. [4] supported our findings and reported that pharmacy students had a generally positive perception of online learning during the COVID-19 pandemic, although they were faced with some challenges, such as a lack of motivation and instructions. Similar findings were also reported by Ambroziak et al. [22]. In this study the students took part in an online survey after using MyDispense and reported “technology difficulties” and “learning how to navigate the program” as the most reported areas for improvement and concluded that whilst time and effort are required to create MyDispense exercises, there is a need to ensure the user can effectively complete them. A systematic review looking at online learning overall for students during the COVID-19 pandemic concluded that only half of the students had positive perceptions of online learning [30]. However, a study focusing on pharmacy students [31] identified they are more comfortable with technology. Continued focus on the technology interfaces to overcome the barriers identified will support the use in the future, along with improvements requested regarding increased resource activity development.

### 4.3. Future Direction

This study looked at MyDispense in relation to dispensing practices; however, there is an opportunity to expand MyDispense usage into responding-to-symptoms (RTS) teaching. Most of the students surveyed believed that these activities would be beneficial for their OSCE preparation. Furthermore, childhood conditions, headaches, respiratory infections, and ear conditions were identified as the preferred topics for future RTS activities using MyDispense. Interestingly, over a third were unsure of any changes that could be made to MyDispense, suggesting that they were satisfied with the system overall. The creation of these activities could assist our students as pharmacy practice transitions from traditional dispensing practices, toward more clinically focused practice. In addition, future exercises could incorporate hospital pharmacy activities. Barisone et al. [32] concluded that students find that web-based simulations deliver the additional support required to learn how to apply concepts in the real world and to better understand the teachings in their curriculum, despite the removal of face-to-face contact. In another study, 98 pharmacy students were surveyed and over 30% reported that would like MyDispense to be incorporated further into their curriculum [26]. Thus, further support to expand the use of MyDispense in pharmacy practice teaching would be provided.

This study has several strengths. First, there was a high participant response rate and thus a larger sample size compared to previous papers, which have focused on the use of MyDispense in pharmacy law and ethics teaching [23]. Furthermore, this paper addresses a gap in the literature by comparing MyDispense usage data to assessment performance data for a range of assessments and not only focusing on exam performance. However, there were some limitations too. This study only focused on one cohort in a COVID-19 pandemic situation. Consequently, these students would have had greater exposure to virtual learning environments and online simulation tools, compared to previous cohorts, and may have had more time to explore MyDispense. There were only four open-ended questions in the survey, and these generated a large range of answers from a small number of students; therefore, overall conclusions cannot be drawn, although the survey provides some useful insights into the perceptions of MyDispense. Future work is required to explore the perceptions of MyDispense in other second-year pharmacy cohorts and other year groups, and to determine the impact of MyDispense usage on exam and coursework performance now that pharmacy teaching has returned to face-to-face. Future initiatives include a focus on continuing to improve accessibility through more tutorial videos and in-person support, designing a broader range of activities, encompassing a wider range of clinical topics, and developing activities for the students to improve their prioritisation skills in a community pharmacy setting.

## 5. Conclusions

MyDispense has provided an accessible supplementary resource to in-person teaching for second-year pharmacy students during the COVID-19 pandemic. Higher levels of MyDispense exercise completion were associated with higher pass rates and mean scores, including a statistically significant association between exercise usage and in-class assessed prescription scores in the “Pharmacy Law, Ethics and Practice” module. Our students found MyDispense to be a beneficial educational tool, which supplemented their learning and supported their revision by enabling them to apply their pharmacy law knowledge and practice their dispensing skills in a ‘safe’ environment.

Using an online system is inclusive as it allows students to complete activities in their own time, which supports their learning. Whilst face-to-face teaching has returned, there remains a place for online additional elements, including MyDispense, in pharmacy teaching.

## Figures and Tables

**Table 1:** What have you enjoyed the most about using MyDispense during this academic year? (*n* = 82).

Response	Number of Students % (*n*=)
MyDispense was accessible from home	12.2% (*n* = 10)
MyDispense provided an opportunity to repeat exercises	9.8% (*n* = 8)
MyDispense was easy to navigate and convenient to use	8.5% (*n* = 7)
MyDispense provided dispensing practice	7.3% (*n* = 6)
MyDispense was realistic and interactive	6.1% (*n* = 5)
MyDispense has helped with revision	6.1% (*n* = 5)
Immediate feedback was provided	3.7% (*n* = 3)
MyDispense provided product selection practice	3.7% (*n* = 3)
MyDispense provided labelling practice	3.7% (*n* = 3)
MyDispense provided a virtual dispensing experience	3.7% (*n* = 3)
MyDispense provided prescription screening practice	2.4% (*n* = 2)
MyDispense helped the students to check the accuracy of their work	2.4% (*n* = 2)
It provided an opportunity to dispense different types of prescriptions	2.4% (*n* = 2)
MyDispense was a good alternative to face-to-face teaching	2.4% (*n* = 2)
MyDispense was essential during the COVID-19 pandemic	2.4% (*n* = 2)
MyDispense mimics community pharmacy practice	1.2% (*n* = 1)
Students were able to replicate conversations with healthcare professionals using MyDispense	1.2% (*n* = 1)
Students were able to create pharmacy records, in addition to dispensing	1.2% (*n* = 1)
Not sure	15.9% (*n* = 13)

**Table 2:** How could MyDispense be improved for future second-year MPharm students? (*n* = 71).

Response	Number of Students % (*n*=)
More instructions to be given prior to using MyDispense	33.8% (*n* = 24)
Layout could be improved, along with software access and user interface	29.6% (*n* = 21)
No improvements needed	8.5% (*n* = 6)
Create more MyDispense exercises	4.2% (*n* = 3)
Incorporate MyDispense into teaching on campus	4.2% (*n* = 3)
Increase the range of products available to dispense	2.8% (*n* = 2)
Create more controlled drug exercises	2.8% (*n* = 2)
Unsure if any improvements are needed	2.8% (*n* = 2)
Fewer, but more detailed, exercises	1.4% (*n* = 1)
Less complex exercises	1.4% (*n* = 1)
Increase the number of exercises with drug interactions	1.4% (*n* = 1)
Increase the number of patient interactions	1.4% (*n* = 1)
Improve the quality of the software	1.4% (*n* = 1)
Add veterinary prescription activities	1.4% (*n* = 1)
Create more record keeping activities	1.4% (*n* = 1)
Provide more feedback for students	1.4% (*n* = 1)

**Table 3:** How could MyDispense be changed to reflect current pharmacy practice? (*n* = 44).

Response	Number of Students % (*n*=)
Not sure	18.1% (*n* = 8)
Not applicable	15.9 % (*n* = 7)
Provide a wider variety and number of MyDispense activities	13.6% (*n* = 6)
Change the dispensing system to a system which is commonly used in community pharmacy	11.4% (*n* = 5)
Create internet pharmacy and hospital pharmacy activities	6.8% (*n* = 3)
Improve the user interface	6.8% (*n* = 3)
Incorporate electronic prescriptions and robot dispensing	4.5% (*n* = 2)
Create a MyDispense app	4.5% (*n* = 2)
Satisfied with current version, no changes are necessary	4.5% (*n* = 2)
Create activities which could be used by qualified pharmacists, not just students	4.5% (*n* = 2)
Add voices for the patients and healthcare professionals	2.3% (*n* = 1)
Develop scenarios for the management of rare diseases	2.3% (*n* = 1)
Offer more MyDispense tutorials	2.3% (*n* = 1)
Make activities compulsory, rather than supplementary	2.3% (*n* = 1)

**Table 4:** Was MyDispense helpful for assessment preparation? (*n* = 115).

Assessment Type	Very Helpful % (*n*=)	Helpful % (*n*=)	Neither % (*n*=)	Not Helpful % (*n*=)	Not Very Helpful % (*n*=)	Missing Data % (*n*=)
Assessed Prescriptions	9.6% (*n* = 11)	36.5% (*n* = 42)	20.9% (*n* = 24)	16.5% (*n* = 19)	8.7% (*n* = 10)	7.8% (*n* = 9)
Mid-module test	5.2% (*n* = 6)	27.8% (*n* = 32)	25.2% (*n* = 29)	22.6% (*n* = 26)	11.3% (*n* = 13)	7.8% (*n* = 9)
Final exam	6.1% (*n* = 7)	27.8% (*n* = 32)	24.3% (*n* = 28)	20.9% (*n* = 24)	13.0% (*n* = 15)	7.8% (*n* = 9)

**Table 5:** Assessment pass rates stratified by MyDispense activity during the academic year (*n* = 147).

Exercises Completed during the Academic Year (20/21)	Passed Assessed Prescription	Passed Mid-Module Test	Passed Final Exam
None	91.3% (*n* = 42/46)	93.5% (*n* = 43/46)	84.8% (*n* = 39/46)
1–10	100% (*n* = 48/48)	93.8% (*n* = 45/48)	91.6% (*n* = 44/48)
11–22	100% (*n* = 37/37)	97.3% (*n* = 36/37)	94.6% (*n* = 35/37)
≥23	100% (*n* = 16/16)	100% (*n* = 16/16)	100% (*n* = 16/16)
*x*^2^ (*df* 3) (*p*)	6.47 (*p* = 0.09)	1.79 (*p* = 0.62)	2.87 (*p* = 0.41)

**Table 6:** Assessment scores stratified by MyDispense activity during the academic year (*n* = 147).

Exercises Completed during the Academic Year (20/21)	Assessed Prescription Mean Score	Mid-Module Test Mean Score	Final Exam Mean Score
None	77.1 (±18.1)	70.7 (±13.0)	65.3 (±15.2)
1–10	83.1 (±12.1)	66.7 (±16.3)	64.9 (±11.6)
11–22	81.2 (±14.9)	67.5 (±13.0)	63.8 (±11.8)
≥23	92.6 (±6.2)	71.9 (±10.5)	70.5 (±8.6)
*F* (*df* = 3) (*p*)	4.53 (*p* = 0.05)	1.01 (*p* = 0.39)	1.09 (*p* = 0.36)

## Data Availability

Not applicable.
